# Curcumin and Nano-Curcumin Mitigate Copper Neurotoxicity by Modulating Oxidative Stress, Inflammation, and Akt/GSK-3β Signaling

**DOI:** 10.3390/molecules26185591

**Published:** 2021-09-15

**Authors:** Wedad S. Sarawi, Ahlam M. Alhusaini, Laila M. Fadda, Hatun A. Alomar, Awatif B. Albaker, Amjad S. Aljrboa, Areej M. Alotaibi, Iman H. Hasan, Ayman M. Mahmoud

**Affiliations:** 1 Pharmacology and Toxicology Department, Faculty of Pharmacy, King Saud University, Riyadh 11451, Saudi Arabia; wsarawi@ksu.edu.sa (W.S.S.); aelhusaini@ksu.edu.sa (A.M.A.); lfadda@ksu.edu.sa (L.M.F.); hetalomar@ksu.edu.sa (H.A.A.); abaker@ksu.edu.sa (A.B.A.); 441203053@student.ksu.edu.sa (A.S.A.); 442204129@student.ksu.edu.sa (A.M.A.); ihasan@ksu.edu.sa (I.H.H.); 2 Department of Pharmacology and Toxicology, College of Pharmacy, Umm Al-Qura University, Makkah 21955, Saudi Arabia; 3 Physiology Division, Zoology Department, Faculty of Science, Beni-Suef University, Beni-Suef 62514, Egypt

**Keywords:** curcumin, GSK-3β, inflammation, DNA damage, oxidative stress

## Abstract

Copper (Cu) is essential for multiple biochemical processes, and copper sulphate (CuSO_4_) is a pesticide used for repelling pests. Accidental or intentional intoxication can induce multiorgan toxicity and could be fatal. Curcumin (CUR) is a potent antioxidant, but its poor systemic bioavailability is the main drawback in its therapeutic uses. This study investigated the protective effect of CUR and N-CUR on CuSO_4_-induced cerebral oxidative stress, inflammation, and apoptosis in rats, pointing to the possible involvement of Akt/GSK-3β. Rats received 100 mg/kg CuSO_4_ and were concurrently treated with CUR or N-CUR for 7 days. Cu-administered rats exhibited a remarkable increase in cerebral malondialdehyde (MDA), NF-κB p65, TNF-α, and IL-6 associated with decreased GSH, SOD, and catalase. Cu provoked DNA fragmentation, upregulated BAX, caspase-3, and p53, and decreased BCL-2 in the brain of rats. N-CUR and CUR ameliorated MDA, NF-κB p65, and pro-inflammatory cytokines, downregulated pro-apoptotic genes, upregulated BCL-2, and enhanced antioxidants and DNA integrity. In addition, both N-CUR and CUR increased AKT Ser473 and GSK-3β Ser9 phosphorylation in the brain of Cu-administered rats. In conclusion, N-CUR and CUR prevent Cu neurotoxicity by attenuating oxidative injury, inflammatory response, and apoptosis and upregulating AKT/GSK-3β signaling. The neuroprotective effect of N-CUR was more potent than CUR.

## 1. Introduction

Copper (Cu) is a redox-active metal found in many organs and tissues. It is essential for a plethora of biochemical processes such as blood clotting, iron absorption, protein homeostasis, energy production, and cellular metabolism [1]. It acts as a cofactor necessary for many redox-regulating proteins [2]. Cu homeostasis is maintained within the normal level by precise regulatory mechanisms that regulate its absorption, excretion, and blood level [3]. Genetic alteration in Cu-regulating ATPases, *ATP7A*, and *ATP7B* can cause Menkes disease (MD) and Wilson disease (WD), respectively [2,4,5]. MD is associated with a defect in Cu absorption and severe Cu deficiency, while WD results in Cu toxicity and affects several organs, including the liver, brain, and eye [2]. Chronic exposure to Cu has been implicated in the pathogenesis of neurodegenerative diseases such as Alzheimer’s disease [6], Parkinson’s disease [7], and familial amyotrophic lateral sclerosis (ALS) [2,8].

Copper sulphate (CuSO_4_) is a well-known pesticide used for repelling pests that decreases the crop yield in agriculture. It is commonly used in tissue culture incubators to minimise the contamination risk as it has bactericidal and fungicidal properties. Accidental or intentional CuSO_4_ intoxication can induce multiorgan dysfunctions that could be fatal. The systemic absorption of Cu occurs through the gastrointestinal tract, lungs, and skin [9]. The clinical manifestations of Cu toxicity are erosive gastropathy, acute liver and kidney injuries, intravascular hemolysis, arrhythmia, rhabdomyolysis, and seizures [10]. Although the mechanisms of CuSO_4_ toxicity are not fully addressed, they represent a combination of significant oxidative stress and endocrine perturbation in the vulnerable organs of the body [11]. Animal studies showed that the chronic oral administration of CuSO_4_ causes liver and kidney functional impairment due to increased Cu levels in the respective organs [12]. The toxic effects of Cu on the liver and kidney have been studied extensively, while the toxicities of other vital organs of the body are less documented. Similar to other metals, the management of Cu toxicity includes the use of chelating agents such as D-penicillamine, tetrathiomolybdate, and trientine [13]. Other chelators such as deferoxamine (DFO) have an affinity for Cu binding [14]. Despite the effectiveness of these chelators, they often associated with some serious adverse effects on cardiovascular, gastrointestinal, respiratory, and nervous systems, which necessitates the use of safer alternatives. In addition, the limited or moderate effectiveness of these chelators has been found in some cases.

Curcumin (CUR) is a hydrophobic polyphenolic compound found natively in turmeric [15]. It exhibits antioxidant [16], antimicrobial [17], anti-inflammatory, pulmoprotective [18], anti-diabetic [19], hepatoprotective [20,21,22], nephroprotective [23], and antitumor actions [24]. In addition to these pharmacological effects, CUR possesses neuroprotective activity where it protected the brain against oxidative injury induced by heavy metals [25]. CUR–cyclodextrin/cellulose nanocrystals (CNCx) exerted more potent antiproliferative effect on prostate and colorectal cancer cell lines than CUR [26]. In addition, CNCx mitigated oxidative stress and improved myelination, and the cellular, electrophysiological, and functional characteristics of Charcot–Marie–Tooth-1A transgenic rats [27]. Recently, Iurciuc-Tincu et al. have immobilized CUR into polysaccharide particles and reported increased stability and bioavailability [28,29]. CUR loading to polysaccharides facilitated overcoming the gastric juice barrier and efficient absorption in the intestine [28,29]. CUR has shown a modulatory effect on glycogen synthase kinase-3 (GSK-3) activity [30], and we have recently reported the involvement of GSK-3β inhibition in mediating its protective efficacy against lead hepatotoxicity [20]. GSK-3β is implicated in neuronal survival; however, the exact mechanism is not clear-cut [31]. Studies have demonstrated increased neuronal death following the overexpression of GSK-3β [32], whereas its knockdown prevents apoptosis [33]. Despite the potent pharmacological effects of CUR, its poor systemic bioavailability and rapid metabolism represent the main drawbacks in its therapeutic uses, which is a problem that was amended by nanoparticle encapsulation [34]. In comparison to the native form, nano-CUR (N-CUR) has a higher solubility and stability but similar activity [15]. Therefore, this nanoformulation can significantly enhance the cell permeability and show more protective effects *in vitro* and *in vivo*. This study was conducted to investigate the involvement of the Akt/GSK-3β pathway in CuSO_4_-induced cerebral oxidative stress, inflammation, and apoptosis in rats and the ameliorative effect of CUR and N-CUR.

## 2. Results

### 2.1. N-CUR and CUR Attenuate Cu-Induced Cerebral Oxidative Stress

**Figure 1:**
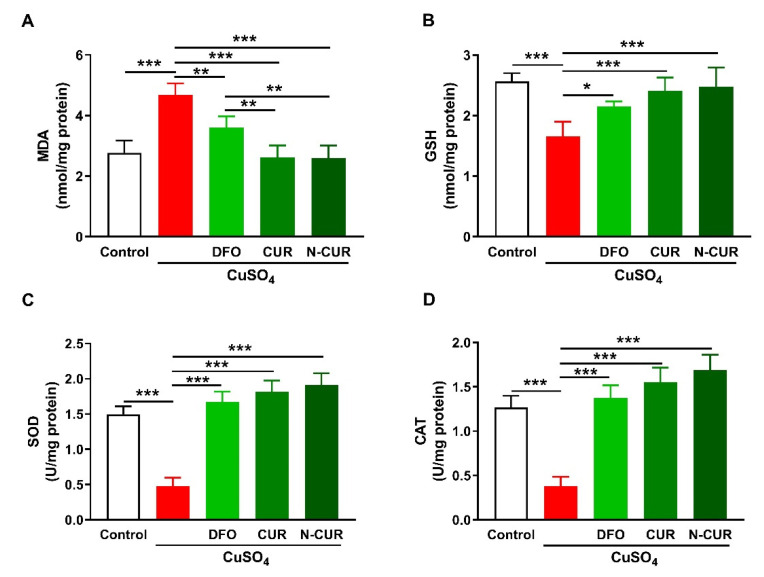
N-CUR and CUR attenuate Cu-induced cerebral oxidative stress. Treatment with N-CUR, CUR, and DFO decreased MDA (**A**) and increased GSH (**B**), SOD (**C**), and CAT (**D**) in the brain of Cu-administered rats. Data are mean ± SEM, (*n* = 8). * *p* < 0.05, ** *p* < 0.01, and *** *p* < 0.001.

The ameliorative effect of CUR and N-CUR on oxidative stress in the brain of Cu-exposed rats was evaluated through the assessment of malondialdehyde (MDA), reduced glutathione (GSH), superoxide dismutase (SOD), and catalase (CAT). Cerebral MDA was significantly elevated in Cu-administered rats when compared with the control group (*p* < 0.001; Figure 1A). In contrast, cerebral GSH content (Figure 1B), SOD activity (Figure 1C), and CAT activity (Figure 1D) were decreased in Cu-administered rats (*p* < 0.001). Treatment with DFO, CUR, and N-CUR decreased MDA and increased GSH, SOD, and CAT in the brain of Cu-administered rats. The effect of both CUR and N-CUR on cerebral MDA was significant compared to DFO (*p* < 0.01).

### 2.2. N-CUR and CUR Suppress Cerebral Inflammation in Cu-Administered Rats

**Figure 2:**
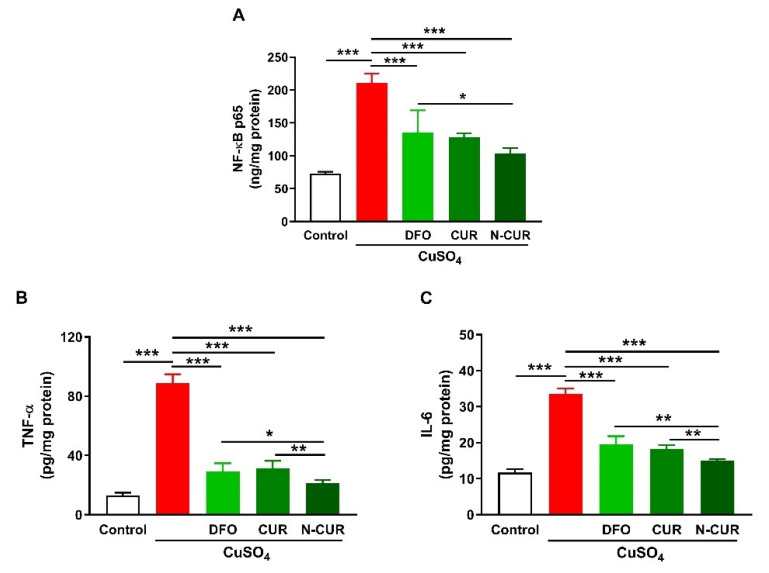
N-CUR and CUR suppress inflammation in Cu-administered rats. Treatment with N-CUR, CUR, and DFO decreased cerebral (**A**) NFκB p65, (**B**) TNF-α, and (**C**) IL-6. Data are mean ± SEM, (*n* = 8). * *p* < 0.05, ** *p* < 0.01 and *** *p* < 0.001.

Cerebral levels of NF-κB p65, TNF-α, and IL-6 were assayed to determine the ameliorative effect of CUR and N-CUR on inflammation induced by Cu ingestion (Figure 2). Cu administration increased NF-κB p65 (Figure 2A), TNF-α (Figure 2B), and IL-6 (Figure 2C) in the cerebrum of rats (*p* < 0.001). All treatments (DFO, CUR, and N-CUR) decreased the levels of cerebral NF-κB p65, TNF-α, and IL-6 significantly (*p* < 0.001). N-CUR was more effective in decreasing cerebral NF-κB p65 (*p* < 0.05) than DFO, and TNF-α, and IL-6 as compared to either DFO or CUR.

### 2.3. N-CUR and CUR Prevent Apoptosis in Cu-Administered Rats

**Figure 3:**
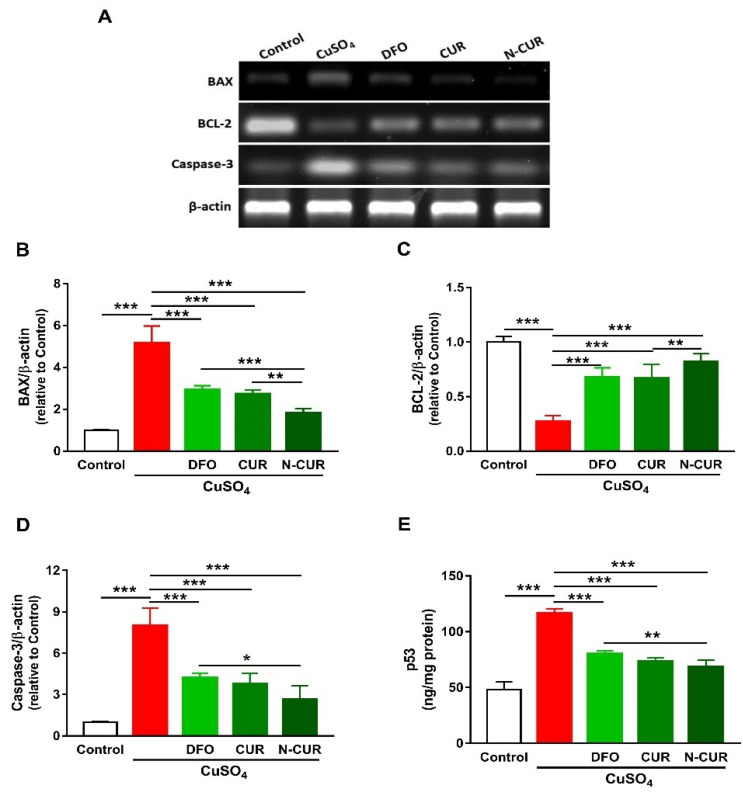
N-CUR and CUR prevent apoptosis in Cu-administered rats. (**A**) Representative blots showing changes in the expression of BAX, BCL-2, and caspase-3. (**B**–**E**) N-CUR, CUR, and DFO decreased (**B**) BAX, increased (**C**) BCL-2, and downregulated (**D**) caspase-3, and (**E**) p53 expression in the brain of Cu-administered rats. Data are mean ± SEM, (*n* = 8). * *p* < 0.05, ** *p* < 0.01 and *** *p* < 0.001.

The expression levels of BAX, caspase-3, and p53 were significantly increased in the cerebrum of rats exposed to Cu as compared to the control group, as depicted in Figure 3. In contrast, rats administered with Cu exhibited a remarkable downregulation of cerebral BCL-2 expression. DFO, CUR, and N-CUR significantly downregulated BAX, p53, and caspase-3 and upregulated BCL-2 in the cerebrum of Cu-administered rats. The effect of N-CUR on BAX and BCL-2 was significant when compared with CUR, whereas its effect was more potent on BAX, caspase-3, and p53 than the effect of DFO.

**Figure 4:**
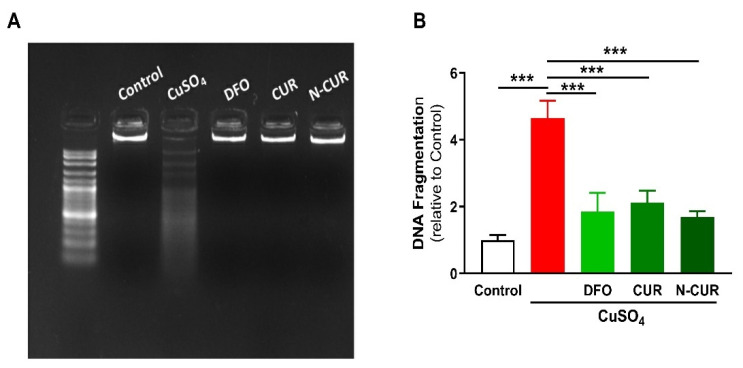
N-CUR, CUR, and DFO prevent DNA fragmentation in the brain of Cu-administered rats. DNA fragmentation was assessed by (**A**) agarose gel electrophoresis and (**B**) colorimetric methods. Data are mean ± SEM, (*n* = 8). *** *p* < 0.001.

The beneficial effects of CUR and N-CUR against Cu-induced cerebral cell death were further confirmed via assessment of DNA fragmentation (Figure 4). Cu-administered rats showed an increase in DNA fragmentation levels as compared to the control group (*p* < 0.001). All treatments (DFO, CUR and N-CUR) prevented the deleterious effect of Cu on DNA integrity.

### 2.4. N-CUR and CUR Upregulate AKT/GSK-3β Signaling in Cu-Administered Rats

**Figure 5:**
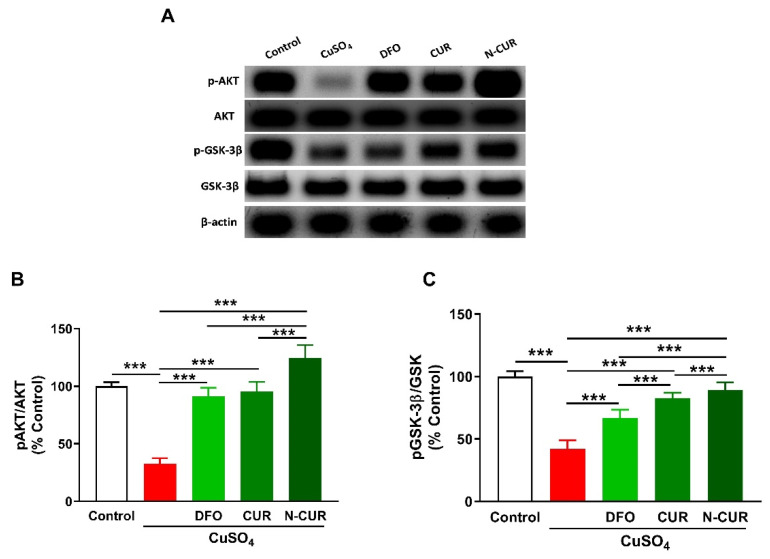
N-CUR and CUR upregulate AKT/GSK-3β signaling in Cu-administered rats. (**A**) Representative blots of pAKT, AKT, pGSK-3β, and GSK-3β. (**B**,**C**) N-CUR, CUR, and DFO increased AKT Ser473 (**B**) and GSK-3β Ser9 (**C**) phosphorylation in the brain of Cu-administered rats. Data are mean ± SEM, (*n* = 8). *** *p* < 0.001.

To investigate the effect of Cu and the ameliorative effect of DFO, CUR, and N-CUR on cerebral AKT/GSK3β signaling, the phosphorylation levels of AKT and GSK3β were determined using Western blotting (Figure 5). Cu-treated rats exhibited a significant decrease in pAKT Ser473 and pGSK-3β Ser9 as compared to the normal rats (*p* < 0.001). Treatment with DFO, CUR, or N-CUR increased cerebral AKT and GSK-3β phosphorylation levels. N-CUR exerted a stronger effect on AKT/GSK-3β signaling than DFO and CUR.

### 2.5. N-CUR and CUR Upregulate Brain-Derived Neurotrophic Factor (BDNF) in Cu-Administered Rats

**Figure 6:**
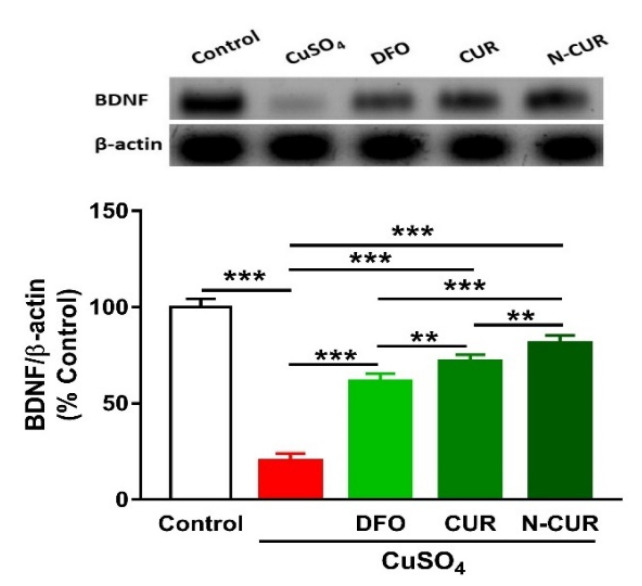
N-CUR and CUR upregulate BDNF in Cu-administered rats. Data are mean ± SEM, (*n* = 8). ** *p* < 0.01 and *** *p* < 0.001.

The administration of Cu resulted in a significant downregulation of BDNF expression in the cerebrum of rats, as shown in Figure 6. Treatment of the Cu-administered rats with DFO, CUR, or N-CUR increased the levels of cerebral BDNF. While the effect of CUR on BDNF was significant as compared to DFO, the effect of N-CUR was more potent when compared to both treatments.

## 3. Discussion

Cu is the third most abundant essential transition metal in humans, and the brain is the second organ containing the highest content after the liver [35]. It is essential for antioxidant defenses, energy homeostasis, and many other physiological processes [1]. However, it may cause neurotoxicity and contribute to the pathogenesis of neurodegenerative diseases [1], where oxidative stress represents the main underlying mechanism [36]. The present results revealed the development of cerebral oxidative stress manifested by elevated MDA and decreased GSH, SOD, and CAT in Cu-administered rats.

Cu cycles easily between stable oxidised and unstable reduced states to coordinate ligands and enzymes and facilitate redox reactions, thereby acting as a cofactor for many enzymes [37]. Although the redox nature makes Cu essential for many biological processes, it renders it toxic because of the generation of highly reactive hydroxyl radicals [36]. In addition, Cu can increase mitochondrial reactive oxygen species (ROS) generation and alter the activity of respiratory chain enzymes [38]. The generated ROS are potent oxidising agents that provoke the oxidative damage of lipids, proteins, and DNA [39], leading to lipid peroxidation (LPO), DNA breaks, and other deleterious effects [40]. Accordingly, LPO was elevated and GSH, SOD, and CAT were declined in the brain of Cu-administered rats in the present study. Given the role of oxidative stress in mediating Cu toxicity, CUR can suppress neurotoxicity via its radical-scavenging and antioxidant properties. Here, rats that received CUR and N-CUR exhibited a remarkable reduction in cerebral MDA levels and enhanced GSH, SOD, and CAT. The antioxidant efficacy of CUR has been reported in numerous studies that employed animal models of neurotoxicity induced by D-galactosamine, fluoride, formaldehyde, rotenone, vincristine, tetrachlorobenzoquinone, pentylenetetrazole, acrylamide, and other agents (reviewed in [41]). In addition, CUR reduced cerebellar LPO in lead-intoxicated rats [25]. These beneficial effects were attributed to the potent radical-scavenging activity of CUR. The activation of nuclear factor erythroid 2-related factor 2 (Nrf2), a redox-sensitive factor that regulates antioxidant genes and suppresses oxidative stress [42], might also have a role in the neuroprotective activity of CUR. In this context, CUR enhanced Nrf2 and antioxidant defenses in rat cerebellar granule neurons challenged with hemin [43] and quinolinic acid-induced neurotoxicity [44].

The upregulation of BDNF in the brain of Cu-administered rats treated with CUR and N-CUR might have a role in boosting the antioxidant defenses through Nrf2 activation. In accordance, a recent study demonstrated that CUR increased BDNF in the brain of quinolinic acid-intoxicated rats, and this activated ERK1/2 and consequently enhanced Nrf2 expression and GSH levels [44]. BDNF belongs to the neurotrophin family and is involved in the maintenance of adult neuronal function [45]. In astrocytes, BDNF has been proposed to play a role in regulating Nrf2 and their metabolic cooperation between neurons [46]. In a model of traumatic brain injury with transplanted neuronal stem cells, BNDF induced Nrf2-mediated antioxidant response [47]. Therefore, this study introduced new information that the upregulation of BDNF plays a role, at least in part, in the protective effect of CUR against Cu neurotoxicity and that N-CUR has a stronger effect on modulating BDNF expression. However, the lack of data showing changes in Nrf2 expression could be considered a limitation of this study.

In addition to the attenuation of oxidative stress, CUR and N-CUR suppressed NF-κB and pro-inflammatory cytokines in the brain of Cu-administered rats. The inflammatory response observed in Cu-administered rats is a direct consequence of excessive ROS generation. The activation of NF-κB and subsequent release of many inflammatory mediators occur as a result of increased cellular ROS [48]. The pro-inflammatory action of Cu is driven by its potential to catalyse ROS generation and decreasing GSH [36], which is an effect reported in the present study. CUR and N-CUR effectively ameliorated cerebral inflammation in Cu-administered rats. N-CUR decreased the levels of TNF-α and IL-6 significantly when compared with CUR, demonstrating enhanced anti-inflammatory activity of the nano form. The ability of CUR to suppress inflammation has been reported in several studies. In a rat model of acrylamide neurotoxicity, Guo et al. [49] showed that CUR attenuated neuroinflammation by decreasing TNF-α and IL-1β levels. In addition, CUR decreased circulating TNF-α in an animal model of lead neurotoxicity [50].

Apoptotic cell death was observed in the brain of Cu-administered rats in the present study. BAX, caspase-3, and p53 were upregulated, whereas the anti-apoptotic BCL-2 was declined in the brain of rats as a result of Cu ingestion. Cu-mediated ROS generation induces mitochondrial permeability transition in astrocytes [51] and hepatocytes [52], leading to cell death via apoptosis. Excess ROS can activate the pro-apoptotic protein BAX, which increases cytochrome *c* release by promoting the loss of membrane potential via mitochondrial voltage-dependent anion channel [53]. Oxidative stress can also provoke p53 nuclear accumulation and its binding to specific DNA sequences, leading to the transcription of genes involved in cell death [54] and the release of mitochondrial cytochrome *c* and the activation of caspases [55]. In contrast, BCL-2 suppresses the release of cytochrome *c* and prevents apoptosis [56]. CUR downregulated the pro-apoptotic factors and increased BCL-2 expression, demonstrating an anti-apoptotic effect that is a direct consequence of its antioxidant and anti-inflammatory properties. The effect of N-CUR on BAX and BCL-2 expression was more potent than CUR. The cytoprotective efficacy of CUR has been reported in a *Drosophila* model of Huntington’s disease [57]. In this model, CUR competently ameliorated neurodegeneration, cytotoxicity, and the compromised neuronal function [57].

To further explore the mechanism underlying the neuroprotective effect of CUR in Cu-administered rats and whether N-CUR is more potent, we determined their effect on AKT/GSK-3β signaling. The phosphorylation of AKT Ser473 and GSK-3β Ser9 was decreased in the brain of Cu-administered rats. While CUR ameliorated the altered phosphorylation levels of these proteins, N-CUR remarkably activated AKT/GSK-3β signaling. Activated AKT mediates the regulation of different processes, including cell growth and proliferation through the phosphorylation of GSK-3, mTOR, NF-κB, and other proteins [58]. AKT controls the activity of GSK-3β, which is active in resting cells, through phosphorylation at Ser9 [59]. Increased GSK-3β activity provoked liver injury [60], whereas its inhibition accelerated the generation of hepatocytes and protected against acetaminophen [61] and lead toxicity [62]. In neuronal cells, the overexpression of GSK-3β induced apoptosis [32,63], demonstrating its crucial role in cell death. BAX phosphorylation has been suggested to be stimulated through GSK-3, and the mutation of GSK-3 inhibited BAX mitochondrial translocation [64]. Moreover, GSK-3 can work in concert with JNK to orchestrate neuronal apoptosis [65]. In the current study, Cu ingestion decreased AKT Ser473 and GSK-3β Ser9 phosphorylation. Reduced inhibition of GSK-3β through its phosphorylation at Ser9 due to suppressed AKT coincides with the observed upregulation of BAX and other mediators of apoptosis. Therefore, the neuroprotective effect of CUR could be directly connected to its ability to activate AKT, which then inhibits GSK-3β-mediated apoptosis. Accordingly, activation of the AKT/GSK-3β signaling by CUR conferred protection against liver injury induced by heavy metals [20]. In support of our findings, computational approaches have demonstrated that CUR inhibits GSK-3β by fitting into its binding pocket [66,67]. This inhibitory effect has been confirmed by an in vitro study showing that the IC_50_ of CUR’s inhibitory activity was 66.3 nM [66]. Furthermore, studies demonstrating the effect of CUR on GSK-3 activity in several diseases have been reviewed by McCubrey et al. [30].

In addition to the findings of this study, Balasubramanian [68,69] presented important quantum chemical insights into the neuroprotective mechanism of CUR and its efficacy to prevent Alzheimer′s disease. The dual property of CUR to be nonpolar in parts and polar in other parts is due to the presence of both phenolic and enolic protons combined with an aliphatic hydrophobic bridge. This property enables CUR to cross the blood–brain barrier (BBB) and bind to and prevent the polymerisation of amyloid-β (Aβ) oligomers [69]. By employing quantum chemical computations, Balasubramanian [68] studied the chelate complexes of CUR with Cu(II) and other transition metal ions that provoke the polymerisation of Aβ and formation of neurotoxic conformations, reporting that the β-diketone bridge, through the loss of an enolic proton of CUR, is the primary site of chelation. CUR can form stable chelate complexes at the β-diketone bridge, thereby scavenging neurotoxic metal ions and inhibiting Aβ polymerisation and the subsequent generation of neurotoxic conformations [68]. Moreover, the ability of piperine, an alkaloid present in black pepper, to enhance the bioavailability and neuroprotective efficacy of CUR is noteworthy of mention. Through the use of quantum chemical and molecular docking, Patil et al. [70] demonstrated that piperine increased the bioavailability of CUR (20-fold) by inhibiting the enzymes mediating CUR glucuronosylation and by intercalating into CUR layers through intermolecular hydrogen bonding [70]. These processes enhance the metabolic transport and consequently the bioavailability of CUR [70]. In support of these findings, Singh et al. [71] reported the protective effect of CUR with piperine, a bioavailability enhancer, against neurotoxicity induced by 3-nitropropionic acid (3-NP) in rats. When supplemented with piperine, CUR improved motor function, attenuated oxidative stress and inflammatory cytokines, and modulated catecholamines and dopamine turnover in the striatum of 3-NP-admninstered rats [71].

## 4. Materials and Methods

### 4.1. Chemicals and Reagents

CuSO_4_, CUR, carboxymethylcellulose (CMC), thiobarbituric acid, agarose, reduced glutathione (GSH), and pyrogallol were obtained from Sigma (St. Louis, MO, USA). Liposomal N-CUR was obtained from Lipolife (Essex, UK), and DFO was purchased from Novartis Pharma AG (Rotkreuz, Switzerland). TNF-α and IL-6 ELISA kits were supplied by R&D Systems (Minneapolis, MN, USA), and the NF-κB p65 ELISA kit was obtained from MyBiosource (San Diego, CA, USA). Antibodies against pAKT Ser473, AKT, pGSK-3β Ser9, GSK-3β, BDNF, and β-actin were supplied by Novus Biologicals (Centennial, CO, USA). Primers were obtained from Sigma (St. Louis, MO, USA). Other chemicals were supplied by standard manufacturers.

### 4.2. Animals and Treatments

Forty male Wistar rats, weighing 180–200 g, were obtained from the Animals Care Centre at King Saud University. The animals were given free access to food and water and acclimatised for one week under standard conditions and 12 h light/dark cycle and free access to food and water. All experimental procedures were conducted in accordance with the requirements of the research ethics Committee at King Saud University (Ethical reference no. SE-19-129). After acclimatisation, the rats were randomly allocated into five groups (*n* = 8) as follows:

- Group I (Control): received 1% CMC orally for 7 days.
- Group II (CuSO_4_): received 100 mg/kg CuSO_4_ dissolved in 1% CMC orally for 7 days [12].
- Group III (DFO): received DFO (23 mg/kg) [72] and 100 mg/kg CuSO_4_ orally for 7 days.
- Group IV (CUR): received 80 mg/kg CUR suspended in 1% CMC [9,72] and 100 mg/kg CuSO_4_ orally for 7 days.
- Group V (N-CUR): received 80 mg/kg N-CUR suspended in 1% CMC [9,72] and 100 mg/kg CuSO_4_ orally for 7 days.

Twenty-four h after the last treatment, the rats were sacrificed under ketamine/xylazine anesthesia. Blood was collected via cardiac puncture and serum was separated by centrifugation. The rats were dissected, and the brain was removed and kept frozen in liquid nitrogen. Other parts from the cerebrum were homogenised in cold PBS (10% *w*/*v*), centrifuged at 5000 rpm for 15 min at 4 °C, and the supernatant was used for assessment of MDA, GSH, SOD, CAT, TNF-α, IL-6, and NF-κB p65.

### 4.3. Determination of MDA and Antioxidants

MDA was determined as previously described [73]. GSH, SOD, and CAT were assayed according to the methods of Ellman [74], Marklund and Marklund [75], and Cohen et al. [76], respectively.

### 4.4. Determination of NF-κB p65, TNF-α, IL-6, and p53

NF-κB p65 was assayed using a specific ELISA kit (MyBioSource, San Diego, CA, USA), and TNF-α and IL-6 were assayed using R&D Systems (Minneapolis, MN, USA) ELISA kits. p53 was determined using ELISA kit supplied by Novus Biologicals (Centennial, CO, USA).

### 4.5. Determination of DNA Fragmentation

Agarose electrophoresis and the colorimetric methods [77] were used to assess DNA fragmentation. The results were presented as a fold change of the control.

### 4.6. Gene Expression

Changes in the expression of BAX, BCL-2, and caspase-3 were determined by RT-PCR as previously described [78]. Briefly, RNA was isolated from the frozen brain samples using TRIzol (ThermoFisher Scientific, Waltham, MA, USA). Following treatment with RNase-free DNase (Qiagen, Hilden, Germany), RNA was quantified using a nanodrop. RNA samples with OD260/OD280 nm ratio of ≥ 1.8 were reverse transcribed into cDNA. The produced cDNA was amplified using PCR master mix (Qiagen, Hilden, Germany) and the primer pairs listed in Table 1. The PCR products were loaded in 1.5% agarose gel, electrophoresed, and the bands were visualised using UV transilluminator. The images were analysed by ImageJ (version 1.32j, NIH, USA), and the values were normalised to β-actin.

### 4.7. Western Blotting

The samples were homogenized in RIPA buffer supplemented with proteinase/phosphatase inhibitors, centrifuged, and protein concentration was determined in the supernatant using Bradford protein assay kit (BioBasic, Markham, Canada). Forty µg protein from each sample was subjected to 10% SDS/PAGE and electrotransferred to nitrocellulose membranes. The membranes were subjected to blocking in 5% milk in TBST followed by incubation overnight at 4 °C with primary antibodies against pAKT Ser473, AKT, pGSK-3β Ser9, GSK-3β, BDNF, and β-actin. The probed membranes were washed, and secondary antibodies were added. After washing, the membranes were washed with TBST, developed using Clarity™ Western ECL Substrate from BIO-RAD (Hercules, CA, USA), and then visualised in ImageQuant LAS 4000. The band intensity was quantified using ImageJ (version 1.32j, NIH, USA).

### 4.8. Statistical Analysis 

The obtained data are expressed as mean ± standard error of the mean (SEM). Statistical analysis was performed by one-way ANOVA and Tukey′s post hoc test using GraphPad Prism 7 (GraphPad Software, San Diego, CA, USA). A *p* value < 0.05 was considered significant.

## 5. Conclusions

These results confer new information on the protective effect of N-CUR on Cu neurotoxicity. N-CUR and CUR attenuated oxidative stress, inflammation, cell death, and oxidative DNA damage in the brain of Cu-administered rats. The modulatory effect of N-CUR and CUR on AKT/GSK-3β signaling was involved, at least in part, in their protective activity against Cu neurotoxicity. The neuroprotective effect of N-CUR was stronger when compared to the native form, which is an effect that could be attributed to the improved properties of CUR.

## Figures and Tables

**Table 1:** Primers used for RT-PCR.

Gene	GenBank Accession Number	Primers (5′–3′)	Product Size (bp)
BAX	NM_017059.2	F: TGGCGATGAACTGGACAACA R: TGTCCAGCCCATGATGGTTC	223
BCL-2	NM_016993.2	F: GAGGGGCTACGAGTGGGATA R: CAATCCTCCCCCAGTTCACC	359
Caspase-3	NM_012922.2	F: GAGCTTGGAACGCGAAGAAA R: GGCAGTAGTCGCCTCTGAAG	472
β-actin	XM_039089807.1	F: CACTCCAAGTATCCACGGCA R: TGCCTCAACACCTCAAACCA	303

## Data Availability

Data analysed or generated during this study are included in this manuscript.

## Sample Availability

Samples of the compounds are not available from the authors.
